# Research on Detection of Sterol Doping in Sports by Electrochemical Sensors: A Review

**DOI:** 10.1155/2022/3394079

**Published:** 2022-09-08

**Authors:** Yunyan Sun

**Affiliations:** Physical Education Department, Nanyang Institute of Technology, Nanyang, Henan 473000, China

## Abstract

The use of doping by athletes to improve performance is prohibited. Therefore, doping testing is an important step to ensure fairness in sports. Doping is gradually metabolized in the body and is therefore difficult to detect immediately by a common method. At the same time, the emergence of new doping agents poses a challenge for highly sensitive detection. Electrochemical sensors are a fast, highly sensitive, and inexpensive analytical detection technology. It provides qualitative and quantitative determination of analytes by altering the electrochemical signal of the analyte or probe at the electrode. In this min-review, we summarized the different electrochemical sensing strategies for sterol doping detection. Some of the representative papers were interpreted in detail. In addition, we compare different sensing strategies.

## 1. Introduction

Doping was originally an opiate mixture for horse racing. The various types of doping banned in sports events include diuretics and inhibitory *β*-blockers. The World Anti-Doping Agency publishes a list of banned drugs every year. There are more than 240 substances explicitly listed in the list of prohibited substances in 2018. Most prohibited substances shall be indicated before use, including but not limited to their metabolites and isomers, as well as other substances with similar chemical structures or biological effects [1–5].

Diuretics can affect the process of urine production through kidneys and other factors and increase the amount of urine discharged by athletes [6–9]. Most of them are used to relieve or eliminate the edema symptoms of patients. Improper use of athletes can achieve the purpose of weight loss. Diuretics rapidly reduce the concentration of other stimulants and their metabolites in body fluids and excreta in a short time, affecting the accuracy of drug test results. Diuretics can interfere with drug testing, resulting in false negative omissions [10–13]. Diuretics and masking agents are high-incidence stimulants. Diuretics can dehydrate athletes, cause muscle spasms, dizziness, fainting, drop blood pressure, and destroy their coordination and balance. Anabolic steroids, also known as anabolic androgenic steroids, have a synthetic structure and biological activity similar to that of testosterone and androstenedione, and are among the most common doping agents in sports. An international survey by the International Olympic Committee showed that 94 (14.8%) of the 634 dietary samples analyzed contained anabolic steroids that were not declared on the label [14]. Synthetic steroids can cross the cell membrane and act directly on the nucleus. Steroids bind to androgen receptors in the nucleus and can increase protein expression, thereby promoting muscle cell growth. Steroids also prevent the breakdown of the muscle tissue caused by stress hormones such as cortisol.

The doping detection methods include gas chromatography, high-performance liquid chromatography, gas chromatography-mass spectrometry, liquid chromatography, mass spectrometry, chemiluminescence immunoassay, and electrochemistry [15–23]. The doping test is closely related to analytical chemistry, especially drug analysis. The emergence of pretreatment methods, separation techniques, and new analytical instruments in analytical chemistry has dramatically improved the ability of doping detection. The advantages and disadvantages of several doping detection methods are compared in Table 1. Because electrochemical detection technology has the advantages of easy miniaturization of the instrument, high sensitivity, rapid analysis, and low detection costs, it is expected to meet the demand for rapid on-site detection.

Sample pretreatment is an essential part of a doping detection. The pretreatment of the sample can prevent the pollution and deterioration of the analytical instrument, prolong the life of the chromatographic column, and improve the sensitivity, accuracy, precision, and selectivity [24–33]. Human urine is the test sample for most doping items. Pretreatment can be divided into protein removal, purification, concentration and enrichment, conjugation hydrolysis, and chemical derivation. The composition of the urine matrix is complex, including uric acid, urea, creatine, trace protein, trace sugar, and endogenous compound metabolites. Due to heavy training and competition, athletes' urine contains more protein than ordinary people, and even hematuria appears. The pretreatment of doping should be combined with the requirements of determination. For different drugs, different pretreatment methods such as enzymatic hydrolysis, separation, purification, and concentration should be selected [34–39].

High-performance capillary electrophoresis (HPCE), developed rapidly based on traditional electrophoresis and chromatography, came out in the early last century. In capillary electrophoresis, ions or charged particles are driven by an electric field. In the capillary, the separation is efficient and fast according to its mobility or distribution coefficient. When a certain voltage is applied to both ends of the capillary, the charged solution moves in the opposite direction of the charge polarity. Because of the different migration speeds of each component in the sample, differential motion is generated. Finally, each component is detected by the detector in order of its speed, while the electrophoretic spectrum of time distribution can be obtained. Capillary electrophoresis technology has many advantages, such as many separation modes, high separation efficiency, fast separation speed, and small sample and reagent consumption [40–43]. It has been widely used in biology, chemistry, environment, medicine, and food analysis [44–48].

The detector is also very important for HPCE. Electrochemical detection includes conductivity detection, amperometric detection, potential detection, and potential gradient detection. Currently, the most commonly used electrochemical detection methods are conductance and amperometric [49–54]. Conductance detection is also called double electrode detection. A constant current is added between the two electrodes, then the potential is measured. Conductance detection is suitable for detecting metal ions and some active ingredients in traditional Chinese medicine, but the main disadvantage of this detector is that the baseline drift and the detection limit are relatively high. The amperometric method is mainly based on the direct ratio of the current generated by the redox reaction on the electrode surface and the analyte concentration [55–59]. This method has been developed in two ways: off-column detection and column end detection.

Tens of thousands of volts of high voltage are applied at both ends of the capillary, making the current in the capillary larger than the detection current by order of magnitude. In order to overcome the influence of a high voltage electric field on electrode detection, the capillary must be divided into the separation part. The interface design shall meet three conditions to allow high pressure to be applied to both ends of the capillary so that the solution and components can enter the detection capillary under the action of electroosmosis [60–64]. The solution or component shall not pass through the interface outside the capillary. The materials used for the interface are nonelectroactive.

Due to the complexity of the fabrication technology, the development of off-column detection technology is restricted. Therefore, the column end amperometric detection is proposed. This method does not need to use the interface to isolate the influence of a high voltage electric field, and the microelectrode is not inserted into the capillary, which also eliminates the interference of electrophoresis current to the detection current.

### 1.1. Capillary Electrophoresis and Electrochemical Methods for Doping Detection

The development of capillary electrophoresis for the analysis of doping began in the 1990s. First, capillary zone electrophoresis and micellar capillary electrochromatography performed some of the earliest work in detecting diuretics by capillary electrophoresis [65, 66]. In the study, when doping and *β*-blockers were added to the urine samples, the solution showed significant UV-absorption peaks [67] or could be detected by laser-induced fluorescence [68]. These studies demonstrated that filtration and centrifugation need to be used for capillary electrophoresis. Subsequently, more sensitive testing methodology began to be used for sample testing, such as capillary zone electrophoresis combines UV absorption and electrospray ionization mass spectrometry. Using instantaneous isokinetic electrophoresis, the sample was sandwiched between buffers with significantly different electrophoretic mobility. This methodology can increase sensitivity by 500 times. Four analytes could be identified in cortisol levels when combined with amperometric testing.

Mannitol is a commonly used doping. Chen et al. [69] proposed capillary electrophoresis and electrochemical detection method to separate and determine mannitol in privet. They studied the effects of NaOH concentration, separation voltage, injection time, and detection potential. In 75 mM sodium hydroxide solution, mannitol can be separated well within 13 min at 12 kV. The relationship between peak current and analyte concentration is linear in three orders of magnitude, and the detection limit of mannitol can be reached to 2 *μ*M.

Hydrochlorothiazid is aslo widely used in antihypertensive preparations to reduce active sodium reabsorption and peripheral vascular resistance. Nezhadaliab and Mojarraba [70] modified a multiwalled carbon nanotube molecularly imprinted polymer onto a graphite electrode and proposed an electrochemical sensor. They chose pyrrole and ethanol as functional monomers and polymeric solvents, respectively. Hydrochlorothiazid has been selected as a template molecule, while carboxyl polywalled carbon nanotubes were electrodeposited on the surface of a pencil-shaped graphite electrode. Then, the molecular imprinting membrane was prepared by electropolymerization of pyrrole. Under optimal conditions, the limit of detection of the proposed sensor is 0.1 nM. This sensor has good reproducibility and reuse ability. Similarly, Alghamdi [71] used anodic stripping voltammetry and cyclic voltammetry to study the electrochemical behavior of hydrochlorothiazid on a glassy carbon electrode. The drug showed a significant volt-ampere oxidation peak at 1.2 V. The electrochemical oxidation process is irreversible and diffusion controlled. The effects of accumulation time, accumulation potential, scanning speed, frequency, and pulse amplitude on the detection were studied. Under optimal conditions, the limit of detection of this method is 4.3 nM. This method does not need to separate or extract samples. Alnajjar et al. [72] reported the first capillary electrophoresis method for the separation and quantification of metoprolol and hydrochlorothiazide. They used a single variable method to optimize the voltage, injection time, and capillary temperature. They also investigated the effects of buffer concentration and pH on separation and detection. The two drugs showed a linear relationship between 2.5 and 250 g/mL in the optimal range. The limit of detection of metoprolol and hydrochlorothiazide were 0.02 and 0.01 g/mL, respectively. Liu et al. [73] evaluated the binding effect of captopril with human serum albumin in the presence of hydrochlorothiazide by capillary electrophoresis. de Carvalho et al. [74] proposed a new method based on ion pair chromatography and pulsed amperometric detection for hydrochlorothiazide, clothalidone, fureuria, and amilolide.

Azzam et al. [75] established a capillary zone electrophoresis method for determining clothalidone. They investigated the effects of buffer pH, concentration, applied voltage, capillary temperature, and injection time on the test. They used phenobarbital as the internal marker and could separate the analyte within 4 min. They also investigated the linear detection range, limit of detection, accuracy, precision, and selectivity. Under optimum condition, the clothalidone can be linear detected between 1 and 250 *μ*g/mL.

Zheng et al. [76] established a simple and effective method for advance capillary electrophoresis detection. Compared with the traditional capillary electrophoresis method, the maximum enhancement factor of the modified method was up to 5500 times. The method was successfully used to determine methylephedrine, selilol, sotalol, and indapamide with the limit of detection of 42 fM, 0.63 pM, 58 fM, and 95 fM, respectively. The relative standard deviation of migration time and peak current for the four analytes was 2% and 3%, respectively.

Li et al. [77] established a capillary zone electrophoresis method for detecting three different formulations of spironolactone. Three kinds of spironolactone showed a good linear relationship in the sulfating *β*-cyclodextrins in phosphate buffer. The method has good accuracy and precision. The analyte recovery was 100.8%∼103.1%. Smajdor et al. [78] recently proposed a new method for the determination of spironolactone by voltammetry. Under optimal conditions, the method can detect the lower spironolactone with a limit of detection of 4.7 nM. The linear detection range of spironolactone is 15 nM–30 *μ*M.

### 1.2. Current Status and Future Outlook

At present, electrophoretic and electrochemical techniques for doping detection need to be further investigated and optimized. The insufficient performance of the doping separation and detection using electrophoresis cannot be very effective in distinguishing different analytes. Although some works have shown that capillary electrophoresis can be used to identify several doping, these techniques have not yet reached the preliminary screening of the samples. A second limitation to the applicability of electrophoresis techniques is the limited sensitivity of the current separation.

However, this does not mean that electrophoresis and electrochemical techniques have no application value in the analysis of doping. The detection sensitivity of electrophoresis has the potential to be further improved. Combining electrophoresis with a range of analytical instruments can overcome this challenge. The emergence of new doping will require continuous improvement of existing technologies. In addition to testing for a single doping, we can focus more on including metabolomics analysis or examining athletes' mitochondria since these markers may be associated with changes at the athlete's cellular level.

## Figures and Tables

**Table 1:** Advantages and disadvantages of several analytical techniques in doping-control.

Instruments	Doping types	Advantages	Disadvantages
UV-vis spectroscopy	With ultraviolet absorption	Cheap instrument	Low sensitivity
GC-MS	Good thermal stability	Qualitative and quantitative analysis	Most of them need to be derived
LC-MS	Good thermal stability	Used for identification of unknown drugs	Structural identification difficulty
Chemiluminescence immunoassay	Sterols and hormones	Sensitivity and specificity	Specific substance detection
Electrochemical method	Electroactive	Sensitivity	Specific substance detection
Gene stimulant transfer vector	Gene doping	Specificity	High cost

## Data Availability

No data were used to support this study.
